# Cardiovascular Disease Knowledge Amongst Jordanian Population: A Cross‐Sectional Study

**DOI:** 10.1002/hsr2.71802

**Published:** 2026-02-26

**Authors:** Ahmed Mohammad Al‐smadi, Salam Bani Hani, Hossam Alhawatmeh, Rula Amr, Omar gammoh, Abedalmajeed Shajrawi, Ala Ashour, Rani Shatnawi., Donna Fitzsimons, Albara Alomari

**Affiliations:** ^1^ Nursing Department Fakeeh College for Medical Sciences, Fakeeh Care Group Jeddah Saudi Arabia; ^2^ Princess Salma Faculty of Nursing Al Al‐Bayt University Mafraq Jordan; ^3^ Faculty of Nursing, Nursing Department Irbid National University Irbid Jordan; ^4^ Adult Health Department College of Nursing, Jordan University of Science and Technology Irbid Jordan; ^5^ Nursing Department Fakeeh College of Medical Sciences Jeddah Saudi Arabia; ^6^ Nutrition and Dietetics Department American University of Madaba Madaba Jordan; ^7^ Faculty of Pharmacy Yarmouk University Irbid Jordan; ^8^ Faculty of Health Sciences Sharjah Campus. Higher Colleges of Technology Academic City UAE; ^9^ Department of Allied Medical Sciences, Faculty of Applied Medical Sciences Jordan University of Science and Technology Irbid Jordan; ^10^ Faculty of Applied Medical Sciences Jordan University of Science and Technology Irbid Jordan; ^11^ School of Nursing and Midwifery Queen's University Belfast Belfast UK; ^12^ College of Health Sciences University of Doha for Science and Technology Doha Qatar

**Keywords:** cardiac symptoms, cardiovascular knowledge, dietary habits, epidemiology, heart attack, Jordan, lifestyle, medical information, nurses, risk factors

## Abstract

**Background and Aim:**

Cardiovascular diseases are the leading cause of death in both developed and developing countries, causing significant healthcare system disruptions due to inadequate access, high costs, and growing complications. This study aims to examine cardiovascular knowledge comprehensively among the Jordanian population.

**Methods:**

A descriptive cross‐sectional design was used to recruit a total of 1,050 participants. A convenience sampling approach was used to gather data from accessible participants who meet the study criteria. A self‐administered questionnaire was used to comprehensively assess participants' knowledge of cardiac diseases composed of 30 questions about dietary knowledge, epidemiology, medical information, risk factors, and heart attack symptoms.

**Results:**

A total of 54.9% were female and 41.0% were employed. The vast majority of participants lived with their families (*n* = 929, 88.5%). Generally, the total scores of all cardiac items have a total score of (Mean = 18.8 out of 30, SD = 1.2), with a mean score of 3.8 out of 6 in dietary knowledge, 2.5 out of 4 in recognizing cardiac epidemiology, 4.0 out of 7 in knowing the cardiac medical information, 6.3 out of 9 in defining the main risk factors to develop cardiac diseases, and only 2.2 out of 4 in identifying the heart attack symptoms. The total score of cardiac knowledge is statistically significantly higher in females compared to males (t [1050] = 2.6, *p* > 0.001). The total score of dietary and cardiovascular risk factors are statistically significant with a higher educational level rather than school and less degree (t [1050] = 3.4, *p* > 0.001, and t [1050] = 2.9 [0.003]), respectively.

**Conclusion:**

Jordan's population has a relatively low public cardiac knowledge score in all areas of heart disease, which may be a result of a lack of health literacy, a lack of educational programs about the further complications of heart disease, and poor socioeconomic conditions among Jordanians.

## 1 Introduction

Cardiovascular diseases (CVD) are viewed as the leading cause of death in developed and developing countries causing devastating effects on the healthcare systems due to inadequate access to healthcare due to the cost and complications that tended to grow sharply among patients and healthcare institutions [1, 2]. Generally, CVD refers to any heart problems affecting cardiac muscle or blood vessels [3]. According to the World Health Organization (WHO), an estimated 17.9 million people died due to heart disease in 2023 representing about 32% of global deaths worldwide [4]. According to the Centers for Disease Control and Prevention (CDC), the top leading cause of death in Jordan was Ischemic Heart Disease (IHD) demonstrating 18% of the total deaths. However, stroke was responsible for 12% of the total death [5].

CVD include several types of health conditions including ischemic heart disease, cerebrovascular disease, peripheral arterial disease, rheumatic heart disease, arrhythmias, congenital heart disease, heart failure, cardiomyopathy, and valvular heart disorders [6, 7]. Sometimes, cardiac disorders could be silent and can be no longer detected with no specific signs and symptoms until the patient experiences a heart attack or heart failure with symptoms including chest discomfort, heartburn, indigestion, shortness of breath, palpitation, swellings in the feet, ankle, and abdomen [8]. Therefore, adequate act for detecting and assessing people at risk of CVD is essential to initiate the primary prevention strategies [2].

The most important risk factors of CVD are preventable since they are modifiable [9]. An unhealthy diet, physical inactivity, and tobacco use are the main risk factors that represent rising blood pressure, lipids, high sugar levels, and being overweight [10]. Besides, about 7 out of 10 people who experience a heart attack have high blood pressure [11]. Around 74.6% of participants had hypercholesterolemia, 37.4% with high blood pressure readings, 32.5% were obese, 31.4% were smokers, and 21.5% with high random blood sugar.

This indicates that risk factors of heart diseases were extremely relevant among the Jordanian population alarming the situation of constant increasing of heart diseases and their complications [12].

Nurses play a crucial role in assessing the public's knowledge of cardiac diseases [13]. They are often on the frontline of healthcare and are in direct contact with patients and the community [14]. Public health initiatives and campaigns in Jordan have focused on raising awareness about CVD risk factors, prevention, and early detection [15]. The Jordanian Ministry of Health, along with various healthcare organizations and non‐governmental organizations, have been working to educate the population about the importance of a healthy lifestyle and regular health check‐ups [16, 17, 18].

It's significant to highlight that people and communities in Jordan can have different levels of public understanding and awareness about CVD. An accurate and current assessment of the population's knowledge and comprehension of CVD in Jordan can be obtained by conducting a cross‐sectional survey. A more knowledgeable population reflects a healthier lifestyle, more recognition of the main risk factors of CVD, and adoption of positive health‐seeking behaviors [19]. Unfortunately, the high burden of CVD among the Jordanian population revealed a limited understanding of the disease knowledge, which is supported by a study that was conducted by Mukattash et al. [20] who found that the general public in Jordan knows nothing about and is unaware of CVD, and they recommend to create and promote efficient and easily available services if they hope to have a beneficial impact on CVD management and prevention. Hence, the purpose of this study is to examine cardiovascular knowledge comprehensively among the Jordanian population.

## 2 Materials and Methods

### 2.1 Study Design

A descriptive cross‐sectional design was used.

### 2.2 Study Population, Sample Size, and Sampling

The study participants were residents of Amman city since it was the highest population density city in Jordan. The sample size was calculated using G*power which shows that the minimum required sample was 1000 participants; based on α = 0.05, power = 0.80, and moderate effect size = 0.30 [21]. However, a larger number of participants were collected to compensate for the attrition rate that could happen during the data collection process. A random sample of different areas in Amman was drawn to form the study cluster. Participants' locations were obtained from the Department of Statistics, which provides free copies of statistical bulletins upon request. They print hundreds of copies of each bulletin, making the data easily accessible. Amman has a total of 4,430,700 population with a density of 584.6 inhabitants/km² which forms 42.0% of the total Jordanian governorate [22]. The statistical bulletins provided by the Department of Statistics (DOS) include detailed demographic information including population size, distribution by district, neighborhood boundaries, and occasionally socioeconomic indicators. These bulletins were used to identify and randomly select different geographic areas within Amman for inclusion in the study cluster. The mention of multiple bulletins being available was intended to highlight the accessibility and comprehensiveness of this data, which supported an unbiased area selection process. In the selected areas, a random sample of 1050 participants was obtained. The inclusion criteria were (1) All Jordanians who were living in Amman, (2) aged more than 18 years, and (3) informed consent on participating in this study. The study was authorized by Al‐al Bayt University Ethics Committee (number: #119/2837/1, date of approval: 4‐1‐2023) and carried out in compliance with the Declaration of Helsinki's standards. Every individual taking part in the study gave their informed consent. After being informed about the purpose and methods of the study.

### 2.3 Data Collection

Data was collected between May and July 2023 by the researchers utilizing a standard predesigned questionnaire for assessing cardiac knowledge among Jordanians [23]. Bilingual professionals translated the instrument from English to Arabic and then reversed it from Arabic to English. Additionally, the researcher tested its accuracy and applicability by having professionals pilot it. A panel of three experts assessed the instrument's content validity, and changes were made as a result.

Demographical data were obtained from the participants including age, sex, educational level, nationality, marital status, living with family, employment status, poor socioeconomic status, presence of chronic diseases, height, and weight to calculate the body mass index (BMI) based on the following formula (kg/m^2^).

Physical activity is measured using the International Physical Activity Questionnaire (IPAQ) – Short Form that classifies the levels of physical activity into three categories, namely, inactive, minimally active, and HEPA active [24], and smoking status.

The questionnaire was developed by Bergman et al. [23]. It consists of 30 questions divided into five primary domains dietary knowledge (six items), epidemiology (four items), medical information (seven items), risk factors (nine items), and heart attack symptoms (four items). The answers were in the form of True/False questions addressing patients' beliefs and knowledge about various aspects of heart disease. If participants were not sure about the correct answer, they were instructed to choose‚ “Don't know.”

For each subdomain and the complete questionnaire, the scoring of the questions was summarized. Each correct answer was given one scoring point. The total score of this questionnaire ranged between 0 and 30.

### 2.4 Data Management and Analysis

Using SPSS software (version 26.0), the gathered data was processed and examined. When presenting quantitative data, regularly distributed continuous variables are represented by mean ± standard deviation. Nominal variables are defined as percentages and absolute numbers (*n*). Inferential statistics, specifically parametric tests, are used to make conclusions about a population based on sample data. A t‐test was utilized to evaluate whether the observed differences were statistically significant. In every analysis, a two‐tailed *p*‐value < 0.001 was considered statistically significant.

## 3 Results

### 3.1 Demographics

The study recruited 1050 participants, where about half of the participants were younger adults aged between 18 and 24 years (*n* = 542, 51.6%). A total of 54.9% were female and 41.0% were employed. The vast majority of participants lived with their families (*n* = 929, 88.5%). approximately, half of the participants had a problem of obesity and overweight 31.5% and 15.7, respectively Table 1.

**Table 1:** Sample charachtristics (*N* = 1050).

Items	*N* (%)
**Age**	
18–25 yrs.	542 (51.6)
26–50 yrs. > 50 yrs.	382 (36.4) 126 (12.0)
**Sex**	
Male	474 (45.1)
Female	576 (54.9)
**Marital status**	
Single	652 (62.1)
Married	398 (37.9)
**Live with family**	
Yes	929 (88.5)
No	121 (11.5)
**Educational level**	
School or less	176 (16.8)
Diploma or higher	874 (83.2)
**Employment status**	
Yes	431 (41.0)
No	619 (59.0)
**Chronic illnesses**	
Yes	135 (12.9)
No	915 (87.1)
**DM**	
Yes	52 (5.00)
No	998 (95.0)
**HTN**	
Yes	72 (6.90)
No	978 (93.1)
**Smoking status**	
Yes	452 (43.0)
No	598 (57.0)
**Physical activity**	
Inactive	851 (81.0)
Minimally active	91 (8.70)
HEPA active	108 (10.3)
**BMI**	
Normal	554 (52.8)
Obese	331 (31.5)
Overweight	165 (15.7)

*Note:* N = number, % = percentages, yrs. = years.

Abbreviations: BMI, body mass index; DM, diabeties mellitus; HTN, hypertention.

### 3.2 Public Knowledge of Cardiovascular Diseases

A summary of participants' scores on the comprehensive cardiac knowledge questionnaire is shown in Table 2. Generally, the total scores of all cardiac items have an average (M = 18.8 out of 30, SD = 1.2), with a total score of 3.8 out of 6 in dietary knowledge, 2.5 out of 4 in recognizing the cardiac epidemiology, 4.0 out of 7 in knowing the cardiac medical information, 6.3 out of 9 in defining the main risk factors to develop cardiac diseases, and only 2.2 out of 4 in identifying the heart attack symptoms.

**Table 2:** Knowledge of public cardiovascular disease among the Jordanian population (*N* = 1050).

Knowledge domain	Min. score	Max. score	Total score ± SD
Cardiac dietary knowledge	0	6	3.8 ± 1.4
Cardiac epidemiology	0	4	2.5 ± 1.1
Cardiac medical information	0	7	4.0 ± 1.6
Cardiac risk factors	0	9	6.3 ± 1.8
Heart attack symptoms	0	4	2.2 ± 1.2
**Total scores of heart knowledge**	**0**	**30**	**18.8** ± **5.3**

Abbreviations: Min. = Minimum, Max. = Maximum, SD = standard deviation.

### 3.3 Demographical Factors Mean Difference in Public Cardiac Knowledge

An independent sample t‐test was used to analyze the mean difference of variables including sex, marital status, living status, educational level, employment status, presence of chronic diseases, and smoking status. Males had significantly lower mean scores than females in diet (18.3 vs. 19.1, *p* > 0.002, 95% CI for the mean difference [female–male]: 0.21–1.49), epidemiology (3.7 vs. 3.9, *p* = 0.042, 95% CI: 0.04–0.39), and medical knowledge (2.3 vs. 2.6, *p* = 0.001, 95% CI: 0.15–0.43). No significant differences were found in risk perception (*p* = 0.525), symptom awareness (*p* = 0.016), or total scores (*p* > 0.587). Married participants scored significantly higher than single participants in risk perception (4.1 vs. 3.9, *p* > 0.001, 95% CI for the mean difference [married–single]: 0.03–0.44). No significant differences were observed in diet, epidemiology, medical knowledge, symptoms, or total scores (*p* > 0.05 for all). No significant differences were found in any category except epidemiology (*p* = *.039*, 95% CI: 0.223–0.323) and medical knowledge (*p* > 0.001, 95% CI: 0.19–0.24), where those not living with family scored slightly higher.

Participants with a diploma or higher had significantly higher scores in Epidemiology (3.9 vs. 3.4, *p* > 0.001, 95% CI: 0.17–0.64) and symptoms knowledge (6.3 vs. 5.9, *p* = 0.003, 95% CI: 0.25–0.78). No significant differences were observed in diet, medical knowledge, risk perception, or total scores. Table 3.

**Table 3:** Demographic factors associated with cardiac knowledge scores (*N* = 1050).

Variables	Total scores	Dietary knowledge	Epidemiology	Medical information	Risk factors	Heart attack Symptoms
	M (SD)	M (SD)	M (SD)	M (SD)	M (SD)	M (SD)
**Sex**						
Male	18.3 (5.7)	3.7 (1.5)	2.3 (1.2)	3.9 (1.7)	6.2 (1.9)	2.2 (1.2)
Female	19.1 (4.9)	3.9 (1.4)	2.6 (1.1)	4.1 (1.6)	6.3 (1.7)	2.3 (1.2)
t‐value (*p‐value*)	2.6 (0.002)	2.4 (0.042)	4.1 (0.001)	92 (0.525)	1.5 (0.016)	1.3 (0.587)
95% CI	1.497–0.210	0.388–0.038	0.425–0.152	0.294–0.106	0.379–0.052	0.237–0.051
**Marital status**						
Single	18.5 (5.2)	3.7 (1.5)	2.5 (1.1)	3.9 (1.6)	6.1 (1.8)	2.3 (1.2)
Married	19.3 (5.4)	3.9 (1.4)	2.5 (1.1)	4.1 (1.7)	6.4 (1.8)	2.2 (1.2)
t‐value (*p*‐value)	2.3 (0.338)	2.5 (0.083)	0.90 (0.792)	2.2 (0.047)	2.6 (0.226)	0.693 (0.937)
95% CI	1.432–0.111	0.409–0.050	0.205–0.076	0.438–0.028	0.096–0.019	0.096–0.199
**Living with family**						
Yes	18.8 (5.3)	3.8 (1.5)	2.5 (1.1)	4.0 (1.6)	6.3 (1.8)	2.2 (1.2)
No	18.6 (5.2)	3.8 (1.3)	2.5 (1.2)	3.9 (1.7)	6.1 (1.6)	2.3 (1.2)
t‐value (*p*‐value)	0.40 (0.688)	0.36 (0.039)	0.21 (0.047)	59 (0.602)	1.2 (0.087)	15 (0.338)
95% CI	0.801–1.21	0.323–0.223	0.237–0.191	0.134–0.537	0.218–0.407	0.241–0.207
**Educational level**						
School or less	17.5 (5.9)	3.4 (1.6)	2.4 (1.1)	3.6 (1.7)	5.9 (2.1)	2.1 (1.3)
Diploma or higher	19.0 (5.1)	3.9 (1.4)	2.5 (1.1)	4.1 (1.6)	6.3 (1.7)	2.3 (1.2)
t‐value (*p*‐value)	3.6 (0.200)	3.4 (0.035)	0.73 (0.572)	3.8 (0.427)	2.9 (0.003)	1.4 (0.084)
95% CI	2.412–0.702	0.639–0.174	0.251–0.115	0.716–0.143	0.779–0.247	0.331–0.523
**Employment status**						
Yes	18.6 (5.3)	3.8 (1.4)	2.4 (1.2)	4.1 (1.7)	6.2 (1.9)	2.1 (1.2)
No	18.9 (5.3)	3.7 (1.5)	2.5 (1.3)	4.0 (1.6)	6.3 (1.7)	2.3 (1.2)
t‐value (*p*‐value)	87 (0.830)	1.0 (0.231)	1.5 (0.742)	52 (0.686)	1.3 (0.100)	2.3 (0.500)
95% CI	0.941–0.365	0.086–0.268	0.247–0.030	0.358–0.078	0.149–0.256	0.329–0.039
**Chronic illness**						
Yes	19.3 (5.5)	3.8 (1.4)	2.6 (1.2)	4.1 (1.7)	6.6 (1.8)	2.2 (1.2)
No	18.7 (5.3)	3.8 (1.5)	2.5 (1.1)	3.9 (1.6)	6.2 (1.8)	2.2 (1.2)
t‐value (*p*‐value)	1.1 (0.376)	0.09 (0.586)	0.69 (0.212)	0.27 (0.531)	2.6 (0.934)	0.12 (0.808)
95% CI	0.423–1.495	0.248–0.273	0.132–0.277	0.103–0.743	0.257–0.339	0.227–0.201
**Smoking status**						
Yes	18.5 (5.3)	3.7 (1.5)	2.4 91.1)	4.0 (1.6)	6.2 (1.8)	2.2 (1.2)
No	18.9 (5.2)	3.9 (1.4)	2.6 (1.1)	3.9 (1.7)	6.3 (1.7)	2.2 (1.2)
t‐value (*p*‐value)	1.1 (0.977)	2.1 (0.056)	1.5 (0.657)	0.14 (0.577)	0.56 (0.420)	0.05 (0.520)
95% CI	1.234–1.727	0.396–0.409	0.134–0.497	0.284–0.706	0.481–0.439	0.462–0.198

Abbreviations: CI, confidence interval; M, mean; SD, standard deviation.

One‐way ANOVA was also run to assess whether differences between groups exist among the BMI and age groups with the total scores of cardiac knowledge. The results found that there are no association among the age groups (F (3, 1047) = 1.19, *p* > 0.001), and for the body mass index, it was (F (3, 1047) = 3.8, *p* > 0.001).

## 4 Discussion

The current study aims to assess the public cardiac knowledge among the Jordanian population using a comprehensive tool that measures different domains including their knowledge about dietary habits, epidemiology of cardiac diseases, risk factors, medical information, and heart attack symptoms. Generally, it could be judged that the Jordanian population had insufficient or poor knowledge about CVDs in all domains of items. The results of this study were consistent with a study conducted by Omoronyia et al. [25], which found that the Iranian population had unsatisfactory knowledge of cardiac diseases and all aspects assessed. Also, another study using the same questionnaire used in this study found poor cardiac knowledge among their participants with poor perception of the main risk factors of CVD and its related risk factors [26]. Poor cardiac knowledge among the participants could be contributing to the lack of awareness about the importance of primary prevention strategies for the complications of CVD among the population. Besides, promoting cardiac knowledge among the population is a crucial priority that could affect the population positively in changing their lifestyle and adopting the healthiest practices which will help in preventing chronic diseases [27].

In this study, females had a higher level of knowledge in all domains of cardiac scores generally with significant results in the aspects of epidemiological and risk factors aspects specifically. In line with this, Omoronyia et al. [25] found that females had a higher cardiac knowledge compared with males and this could be contributing that females are more interested in getting health information that obtained from different sources of information including their frequent visiting maternity clinics and more access to health care services rather than males. Furthermore, this study found that participants with higher educational levels had a higher cardiac knowledge which is consistent with a study that was conducted by Chukwuemeka et al. [28] who revealed that knowledge of cardiac risk factors is increased with increasing their educational level.

The current study showed that there is no significant result between the body mass index of participants and the total score of cardiac knowledge which is inconsistent with a study performed by Michaëlsson et al. [29, 30]who outlined that obese individuals did not experience higher cardiac knowledge scores while those who have normal BMI adhere to a healthy diet program to maintain a low risk of mortality.

### 4.1 Strengths and Limitations of the Study

The study has limitations even though it has a large sample size and a generally homogeneous socioeconomic setting across different Jordanian governorates, which increases the findings' potential generalizability. First, it is difficult to prove causality or shifts in knowledge over time due to the cross‐sectional design. Second, there may have been selection bias since those who consented to participate may have been better educated or more health‐conscious than the general population, which could have resulted in an overestimation of knowledge about CVD. Furthermore, although using a thorough CVD knowledge questionnaire improves the evaluation, self‐reported answers could be skewed by social desirability or recollection bias. Furthermore, even though this is the first study in Jordan that has been recognized to thoroughly evaluate public understanding of CVD, the results should be interpreted cautiously when extrapolating to populations with alternative cultural or health system contexts.

### 4.2 Implication of Study

Nursing professionals serve as educators and public health activists since they can take part in community outreach initiatives, health fairs, and workshops where they disseminate knowledge about cardiac illnesses, risk factors, management, and prevention. Besides, collaboration with other healthcare providers is another implication to coordinate complete treatment for people with cardiac problems because nurses work closely with doctors, dietitians, and other healthcare providers. Nurses work with these professionals to produce and provide accurate, scientifically sound instructional materials in a public context.

## 5 Conclusion

It is concluded that the Jordanian population has a low public cardiac knowledge score in all aspects of heart diseases including dietary habits, epidemiology of cardiac diseases, risk factors, medical information, and heart attack symptoms. This could be attributed to the lack of educational programs about the further complications of CVD, poor health literacy, poor socioeconomic conditions among the Jordanian population, and lack of system training programs to educate the general population.

## Author Contributions

**Ahmed Mohammad Al‐smadi:** conceptualization, formal analysis, writing – original draft. **Salam Bani Hani:** data curation. **Hossam Alhawatmeh:** data curation. **Rula Amr:** data curation. **Omar gammoh:** writing – original draft, writing – review and editing, supervision, formal analysis. **Abedalmajeed Shajrawi:** writing – review and editing, writing – original draft. **Ala Ashour:** writing – review and editing. **Rani Shatnawi:** data curation, investigation. **Donna Fitzsimons:** writing – review and editing. **Albara Alomari:** visualization, project administration, writing – review and editing.

## Funding

The authors received no specific funding for this work.

## Conflicts of Interest

All authors declare no conflicts of interest.

## Transparency Statement

The lead author Albara Alomari affirms that this manuscript is an honest, accurate, and transparent account of the study being reported; that no important aspects of the study have been omitted; and that any discrepancies from the study as planned (and, if relevant, registered) have been explained.

## Data Availability

The data that support the findings of this study are available upon reasonable request.

## References

[1] A. Oyeyemi and P. Scott , “Interoperability in Health and Social Care: Organisational Issues are the Biggest Challenge,” BMJ Health and Care Informatics 25 (2018), 10.14236/jhi.v25i3.1024. 30398464

[2] S. H. Bani Hani and M. M. Ahmad , “Machine‐Learning Algorithms for Ischemic Heart Disease Prediction: A Systematic Review,” Current Cardiology Reviews 19 (2023): 87–99. 10.2174/1573403X18666220609123053 PMC10201879 35692135

[3] K. Mc Namara , H. Alzubaidi , and J. K. Jackson , “Cardiovascular Disease as a Leading Cause of Death: How are Pharmacists Getting Involved?,” Integrated Pharmacy Research and Practice 8 (2019): 1–11. 30788283 10.2147/IPRP.S133088 PMC6366352

[4] WHO . 2023. Cardiovascular Diseases.

[5] CDC . 2021.

[6] M. Hamer , G. O'donovan , and E. Stamatakis , “Association Between Physical Activity and Sub‐Types Of Cardiovascular Disease Death Causes in a General Population Cohort,” European Journal of Epidemiology 34 (2019): 483–487. 30417220 10.1007/s10654-018-0460-2 PMC6456476

[7] S. K. Masenga and A. Kirabo , “Hypertensive Heart Disease: Risk Factors, Complications and Mechanisms,” Frontiers in Cardiovascular Medicine 10 (2023): 1205475. 37342440 10.3389/fcvm.2023.1205475 PMC10277698

[8] M. H. Seo , W.‐Y. Lee , S. S. Kim , et al., “2018 Korean Society for the Study of Obesity Guideline for the Management of Obesity in Korea,” Journal of Obesity and Metabolic Syndrome 28 (2019): 40–45. 31089578 10.7570/jomes.2019.28.1.40 PMC6484940

[9] K. F. Alhabib , M. A. Batais , T. H. Almigbal , et al., “Demographic, Behavioral, and Cardiovascular Disease Risk Factors in the Saudi Population: Results From the Prospective Urban Rural Epidemiology Study (Pure‐Saudi),” BMC Public Health 20 (2020): 1213. 32770968 10.1186/s12889-020-09298-w PMC7414714

[10] B. Nowrouzi‐Kia , A. K. C. Li , C. Nguyen , and J. Casole , “Heart Disease and Occupational Risk Factors in the Canadian Population: An Exploratory Study Using the Canadian Community Health Survey,” Safety and Health at Work 9 (2018): 144–148. 29928527 10.1016/j.shaw.2017.07.008 PMC6005906

[11] CDC . 2020. Heart Disease and Stroke, https://www.Cdc.Gov/Chronicdisease/Resources/Publications/Factsheets/Heart‐Disease‐Stroke.Htm.

[12] W. S. Alsaud , M. Tabbaa , V. Kasabri , et al., “Prevalence Of Cardiovascular Diseases Risk Factors Among Jordanians,” Journal of the Saudi Heart Association 32 (2020): 324–333. 33154938 10.37616/2212-5043.1074 PMC7640553

[13] D. Richter , L. Guasti , D. Walker , et al., “Frailty In Cardiology: Definition, Assessment and Clinical Implications For General Cardiology. A Consensus Document Of The Council For Cardiology Practice (Ccp), Association For Acute Cardio Vascular Care (Acvc), Association Of Cardiovascular Nursing and Allied Professions (Acnap), European Association Of Preventive Cardiology (Eapc), European Heart Rhythm Association (Ehra), Council on Valvular Heart Diseases (Vhd), Council on Hypertension (Cht), Council Of Cardio‐Oncology (Cco), Working Group (Wg) Aorta and Peripheral Vascular Diseases, Wg E‐Cardiology, Wg Thrombosis, Of The European Society Of Cardiology, European Primary Care Cardiology Society (Epccs),” European Journal of Preventive Cardiology 29 (2022): 216–227. 34270717 10.1093/eurjpc/zwaa167

[14] S. B. Hani , K. M. Aldiabat , and M. A. Qadire , “Nursing Leadership Style, Training Methods, and Use of Electronic Health Records by Nurses In Jordanian Hospitals: A Descriptive Study,” Florence Nightingale Journal of Nursing 30 (2022): 110–116. 35699627 10.54614/FNJN.2022.20177 PMC9449718

[15] A. R. Saifan , I. A. Alarabyat , I. Alrimawi , and N. Al‐Nsair , “Utilizing Telehealth Intervention to Support Patients With Cardiovascular Diseases in Jordan: A Qualitative Study,” Applied Nursing Research 68 (2022): 151641. 36473721 10.1016/j.apnr.2022.151641

[16] O. Al‐Hadeethi , M. Al Nsour , Y. Khader , O. K. Alkhlaifat , H. Al Jawaldeh , and A. Hayajneh , “The Capacity of Primary Health Care Centers in Jordan to Manage Hypertension: Areas for Improvement,” Journal of Human Hypertension 36 (2022): 473–481. 33106597 10.1038/s41371-020-00433-z

[17] W. H. Organization 2020. Hearts: Technical Package For Cardiovascular Disease Management In Primary Health Care.

[18] D. R. J. Collins , K. Jobanputra , T. Frost , et al., “Cardiovascular Disease Risk and Prevention Amongst Syrian Refugees: Mixed Methods Study of Médecins Sans Frontières Programme in JORDAN,” Conflict and Health 11 (2017): 14. 28725259 10.1186/s13031-017-0115-z PMC5512828

[19] R. Ndejjo , F. Nuwaha , H. Bastiaens , R. K. Wanyenze , and G. Musinguzi , “Cardiovascular Disease Prevention Knowledge and Associated Factors Among Adults in Mukono and Buikwe Districts in Uganda,” BMC Public Health 20 (2020): 1151. 32698818 10.1186/s12889-020-09264-6 PMC7374818

[20] T. L. Mukattash , M. Shara , A. S. Jarab , S. I. Al‐Azzam , A. Almaaytah , and Y. N. Al Hamarneh , “Public Knowledge and Awareness Of Cardiovascular Disease and Its Risk Factors: A Cross‐Sectional Study Of 1000 Jordanians,” International Journal of Pharmacy Practice 20 (2012): 367–376. 23134095 10.1111/j.2042-7174.2012.00208.x

[21] F. Faul , E. Erdfelder , A. Buchner , and A.‐G. Lang , “Statistical Power Analyses Using G* Power 3.1: Tests For Correlation and Regression Analyses,” Behavior Research Methods 41 (2009): 1149–1160. 19897823 10.3758/BRM.41.4.1149

[22] DOS . 2019. Jordan In Figures 2019.

[23] H. E. Bergman , B. B. Reeve , R. P. Moser , S. Scholl , and W. M. P. Klein , “Development of a Comprehensive Heart Disease Knowledge Questionnaire,” American Journal of Health Education 42 (2011): 74–87. 21720571 10.1080/19325037.2011.10599175 PMC3124098

[24] G. Lavelle , M. Noorkoiv , N. Theis , et al., “Validity Of The International Physical Activity Questionnaire Short Form (Ipaq‐Sf) as a Measure Of Physical Activity (Pa) in Young People With Cerebral Palsy: A Cross‐Sectional Study,” Physiotherapy 107 (2020): 209–215. 32026822 10.1016/j.physio.2019.08.013

[25] O. Omoronyia , A. Ayuk , K. Nwafor , and A. Legogie , “Hypertensive Patients' Knowledge Of Cardiovascular Disease in Calabar, Nigeria,” Journal of the Egyptian Public Health Association 95 (2020): 16. 32813101 10.1186/s42506-020-00045-y PMC7364754

[26] P. Ezzati and S. Salehi , “Correlation Of Heart Knowledge and Cardiac Risk Factors With Readiness For Lifestyle Modification in Companions of Patients With Cardiovascular Diseases in the West Of Iran,” Middle East Journal of Rehabilitation and Health 6 (2019): e88622, 10.5812/mejrh.88622.

[27] H. Sheikh Sharafi and B. Seyed Amini , “Assessment Of Health Literacy and Self‐Care In Heart Failure Patients,” Journal of Health Literacy 1 (2017): 203–219.

[28] U. M. Chukwuemeka , F. C. Okoro , U. P. Okonkwo , et al., “Knowledge, Awareness, and Presence of Cardiovascular Risk Factors Among College Staff of a Nigerian University,” Bulletin of Faculty of Physical Therapy 28 (2023): 8.

[29] K. Michaëlsson , J. A. Baron , L. Byberg , et al., “Combined Associations of Body Mass Index and Adherence To A Mediterranean‐Like Diet With All‐Cause and Cardiovascular Mortality: A Cohort Study,” PLoS Medicine 17 (2020): e1003331. 32941436 10.1371/journal.pmed.1003331 PMC7497998

[30] A. Pandey , M. Lamonte , L. Klein , et al., “Relationship Between Physical Activity, Body Mass Index, and Risk Of Heart Failure,” Journal of the American College of Cardiology 69 (2017): 1129–1142. 28254175 10.1016/j.jacc.2016.11.081 PMC5848099

